# PrEPared to choose: the protocol for an implementation study of the delivery of cabotegravir long-acting injectable pre-exposure prophylaxis (PrEP) as an HIV prevention product option within a real world PrEP choice context in cape town

**DOI:** 10.1186/s12889-025-24370-z

**Published:** 2025-09-24

**Authors:** C. Pike, E. Rousseau, P. Macdonald, P. Mapukata, K. Lebelo, D. Joseph-Davey, F. Little, L. G. Bekker

**Affiliations:** 1 Faculty of Health Sciences, Desmond Tutu HIV Centre, Institute of Infectious Disease and Molecular Medicine, University of Cape Town, Cape Town, South Africa; 2 Division of Infectious Diseases, David Geffen School of Medicine, University of California Los Angeles, Los Angeles, United States of America; 3 Department of Epidemiology and Biostatistics, School of Public Health, University of Cape Town, Cape Town, South Africa; 4 Department of Statistical Sciences, University of Cape Town, Cape Town, South Africa

**Keywords:** HIV prevention, Injectable PrEP, PrEP choice, Adolescents and young people, South africa

## Abstract

**Background:**

Increasing choice among HIV pre-exposure prophylaxis (PrEP) products bears potential to increase uptake, persistence, and coverage amongst those at risk of HIV acquisition. Few studies have evaluated PrEP persistence during real-world delivery of multiple PrEP products from community-based sites to adolescents and young people.

**Methods:**

The PrEPared to Choose (PtC) study delivers PrEP choice across oral, injectable, and vaginal ring options to adolescents and young people (15–29 years) and their potential male partners in Cape Town, South Africa. This phase 3B clinical trial utilizes a type 2 hybrid implementation design with co-primary clinical and implementation aims that include an analysis of PrEP persistence (defined as sustained use of PrEP product as intended with < 7 vs. < 28 day gap in dosing as scheduled) over the short term (7 months) and long term (18 months), and the identification of implementation strategies that best support PrEP adoption and persistence. PtC is delivered from a mobile clinic and a public health primary care clinic, staffed by trained nurses, HIV counsellors, and peer-navigators. PrEP selection is guided by a co-created PrEP choice counselling intervention, with allowance for product switching at subsequent visits, but no reimbursement for PrEP uptake or return.

**Discussion:**

PrEPared to Choose will provide an early report of real-world PrEP choice delivery, including all three currently available and approved modalities. The protocol is designed to simulate a real-world environment that provides insight into likely PrEP persistence patterns and practical challenges to PrEP choice implementation in a high HIV burden setting (South Africa) and within high HIV incidence populations (adolescents and young people). The results will be used to inform PrEP choice delivery in South Africa and build a framework into which future, emerging PrEP modalities can be incorporated.

**Trial approvals and registration:**

This is a phase 3B clinical trial registered with the South African Health Products Regulatory Authority (20230904). Ethical approval was granted by the Human Research Ethics Committee (Faculty of Health Sciences, University of Cape Town, 567/2023). It is registered with the South African National Clinical Trial Registry (DOH-27-012024-5189, 26 January 2024) and ClinicalTrials.gov (NCT06807736, retrospectively registered on 4 February 2025).

## Background

HIV pre-exposure prophylaxis (PrEP) formulations have expanded in recent years, moving from a single daily, oral tablet option to include a monthly intra-vaginal ring (DapiRing) and a two-monthly intramuscular injection (Cabotegravir long-acting, CAB LA), with further long-acting options imminently anticipated [1–4]. As occurred with contraceptive choice, the introduction of PrEP choice is expected to increase PrEP uptake, persistence, and ultimately HIV exposure coverage. This is predominantly because new formulations stand to mitigate challenges with daily adherence and pill burden previously reported by oral PrEP users [1, 5]. 

South Africa (SA) has one of the largest HIV epidemics globally, with 5.8 million people on treatment and 149 000 new infections in 2023 [6]. The benefits of primary HIV prevention are both to the individual, who avoids a lifetime of treatment and HIV-associated sequelae, and the state that must otherwise fund an expensive HIV treatment and care programme. Increasing PrEP coverage requires an increased product uptake as well as increased persistent use, both of which have been historically sub-optimal when oral PrEP alone was available, despite high efficacy, safety, and widespread availability in local SA primary care clinics to anyone that perceives themselves at risk of HIV infection [1, 3]. 

The burden of new HIV infections in SA is primarily amongst adolescents and young people, particularly adolescent girls and young women and gender and sexually diverse populations [7, 8]. There is, however, increasing acknowledgment of the need to engage male partners, who have historically shown low uptake of HIV testing and prevention interventions [9]. Men, adolescents, pregnant and breastfeeding women, and young people have historically shown poor uptake of oral PrEP, yet indicate a theoretical preference for long-acting formulations and so stand to benefit from expanded PrEP options [10–15]. 

The REACH study investigated adherence to oral PrEP and the DapiRing amongst sub-Saharan African adolescent girls and young women [16]. This open-label study showed higher adherence to oral PrEP and DapiRing compared to previous trials, with most participants preferring the longer-acting DapiRing due to its lower user burden [16]. This provides support for the hypothesis, to be tested in the ongoing PrEPared to Choose (PtC) study, that expanding PrEP choice may improve PrEP adherence and ultimately increase HIV prevention coverage to reduce overall HIV acquistion [16]. With the DapiRing and a two-monthly injectable PrEP (Cabotegravir long-acting, CAB LA) approved for HIV prevention in SA for in 2022 [16, 17], there is an urgent need to deliver PrEP choice to adolescents, young people, and their intimate partners, in a manner that builds on the lessons of oral PrEP implementation and that reaches the populations most likely to benefit.

## Study design

PtC seeks to describe patterns of persistence of HIV PrEP products, individually and overall, amongst a cohort of South African adolescents and youth (aged 15–29 years) when offered different PrEP modalities (oral vs. DapiRing vs. injectable) within a sexual and reproductive health service (SRH) package and supported by comprehensive choice counselling. PtC will probe implementation barriers and enablers in PrEP adoption (decision to use any PrEP product), initiation (first dose of any PrEP product), and persistence amongst both PrEP users and PrEP providers (specialised nurses and HIV counsellors) in real-world government primary health care facilities and mobile clinic settings. PtC utilises a hybrid implementation design with co-primary implementation and clinical aims, in the form of a non-randomised, quasi-experimental trial design.

### Aims and objectives

The primary clinical aim of PtC is to describe patterns of short and long-term persistence (as measured over 7 months and 18 months respectively) across three HIV PrEP products, individually and overall, amongst a cohort of South African adolescents and youth. Participants are offered a choice between PrEP products, following HIV testing and comprehensive choice counselling. PrEP products include (a) oral PrEP (Emtricitabine/tenofovir), prescribed as a daily tablet or for event-driven use for select male participants; (b) a Dapivirine-containing vaginal ring (DapiRing) that requires monthly replacement, for non-pregnant and non-breastfeeding adolescent girls and young women; and (c) Cabotegravir long-acting (CAB-LA) as a two monthly intra-muscular gluteal injection.

PrEP persistence is defined as sustained, continuous use of the selected product as intended, with consideration of both (a) a < 7-day gap in PrEP availability for daily dosing (oral PrEP), DapiRing insertion, or subsequent injection or (b) a < 28-day gap. The 7-day gap between the scheduled return vs. actual return is consistent with the definitions used in CAB-LA phase 3 clinical trials [17]. – [18] The 28-day gap follows South African recommendation guidelines for a “missed visit” and is the clinical time point where re-initiation on CAB-LA would be needed. In the South African context, it is expected that the 28 day gap will provide a more realistic timeframe for return. In addition to a missed visit, non-persistence on a specific product is further defined as any participant that switches to using another PrEP product with (a) < 7 days and (b) < 28-day gap between products. PrEP persistence in the cohort, by product and on PrEP overall, will be calculated and reported at 7 months (short-term persistence) and 18 months (long term persistence). The comparison of PrEP persistence by product will be related to the hypothesis that there will be a significantly greater persistence in the CAB LA group compared to oral PrEP and/or DapiRing.

The primary implementation aim is to determine what combination of implementation strategies best achieve PrEP adoption (decision to use any PrEP product), active implementation (PrEP initiation), and persistence (sustained PrEP use) outcomes within a RE-AIM (Reach, Effectiveness, Adoption, Implementation, Maintenance) implementation science framework. This will be assessed over the short term (7 months) and long term (18 months), considering barriers and enablers for both the PrEP user and PrEP provider. Mixed qualitative and quantitative methods and process evaluations will be used to inform the implementation analysis.

Several secondary aims and an exploratory aim are described in Table 1.

**Table 1:** An overview of PtC study primary and secondary aims, including their endpoints and time of measurement

Aim	Endpoint (s)	Time of measurement
*Primary Aims*		
1.1 To compare short term persistence (over 7 months) across PrEP modalities and PrEP overall (*Clinical aim)*	Persistence - defined as sustained, continuous use of the selected PrEP product as intended, with consideration of both (a) a < 7 day gap or (b) a < 28 day gap in PrEP provision for daily dosing (oral PrEP), DapiRing insertion, or subsequent injection. Participants that product switch are considered *PrEP persistent overall* but not *PrEP product persistent*. Product use patterns, including product switches and re-starts, are tracked for the full 7-month period for all persistent and non-persistent participants.	7 months from PrEP initiation.
1.2 To determine what combination of implementation strategies best achieve PrEP adoption and persistence over the short term within a RE-AIM framework (*Implementation aim).*	*Reach* – (i) time to recruit the injectable PrEP study population (*n* = 900 participants); (ii) demographic and clinical characteristics of PrEP initiators vs. persistent users vs. non-persistent users in each PrEP product user group. *Effectiveness* – (i) frequency and description of PrEP product reported side effects; (ii) frequency and description of reported social harms, including intimate partner violence. *Adoption* – (i) proportion of participants reached and sustained at mobile clinic vs. government facilities; (ii) frequency, timing, and nature of product switches and PrEP discontinuation, informed by a qualitative assessment of reasons for non-persistence. *Implementation* – (i) description and evaluation of PrEP choice counselling delivery as per counselling time per person, fidelity assessment scores, and correlation of documented counselling outcomes with actual PrEP persistence; (ii) Evaluation of injectable PrEP administration according to clinical operating procedures. *Sustainability* – (i) sustainability of PrEP choice delivery at participating sites as assessed through the clinical sustainability assessment tool and (ii) qualitative assessment of acceptability and readiness to receive future long-acting PrEP products.	All participants having passed through (a) 7 months and (b) 18 months of study participation.
*Secondary Aims*		
2.1 To compare long term persistence (over 18 months) across PrEP modalities and PrEP overall (*Clinical aim)*	As above for aim 1.1.	18 months from PrEP initiation.
2.2 Time on PrEP based on expected (injections given; product collected) vs. actual (self-reported patterns of use) PrEP coverage *(Clinical aim*)	Total time on PrEP overall based on oral/vaginal PrEP collection and injections vs. self-reported patterns of use. Actual time and self-reported time on PrEP for injectable PrEP users is equivalent.	7 months and 18 months from PrEP initiation.
2.3 Safety, tolerability and acceptance of all three methods *(Clinical aim*)	Safety – frequency of reported adverse events and serious adverse events, including maternal and infant outcomes in pregnant and breastfeeding populations. Tolerability – frequency of self-reported side effects by PrEP method and relation to PrEP non-persistence. Acceptance – reasons for PrEP and PrEP product persistence or discontinuation, as reported at PrEP study visits, retention calls, PrEP user surveys, and PrEP user in-depth interviews.	7 months and 18 months from PrEP initiation.
2.4 To evaluate the sensitivity and specificity of counsellor-administered rapid Antigen/Antibody (Ag/Ab) HIV tests compared to NAAT testing results in an injectable PrEP-using population (*Clinical aim)*	All participants undergo three HIV tests at the specified time points to allow for comparison of HIV test results from (a) counsellor administered 3rd generation rapid antibody (Ab) HIV tests; (b) counsellor administered 4th generation rapid Ag/Ab HIV tests; vs. (c) HIV RNA polymerase chain reaction (HIV viral load) tests sent to an independent laboratory.	Baseline, month 1, month 7 (range 5–6 months), and month 18 (range 16–18 months, at study exit).
*Exploratory aim*		
3.1 HIV incidence across PrEP modalities	The study sample size has insufficient power to allow for formal analysis of HIV incidence. HIV incidence by PrEP modality will be described and compared. Viral resistance testing will be conducted for all participants that seroconvert while on any PrEP product.	7 months and 18 months from PrEP initiation

### Study population and setting

#### PrEP users

PtC utilises a prospective cohort design that recruits up to 1800 adolescents and young people, specifically adolescent girls and young women (15–29 years) including pregnant and breastfeeding women, young male key populations including gay and other men who have sex with men (15–29 years), and their collective male partners (> 15 years). In order to participate, the participant must belong to one of three population groups as described, be HIV negative at baseline, be resident in or have frequent presence within the study area, and able to provide voluntary informed consent to participate in the study. The study aims to recruit at least 100 pregnant and breastfeeding participants. Use of a particular PrEP product may be further restricted by clinical criteria, which differs by product (see Table 2).

**Table 2:** Exclusion criteria by product

	Tenofovir disoproxil and emtricitabine (Oral PrEP)	Dapivirine (Vaginal Ring)	Cabotegravir long-acting (Injectable PrEP)
	Unknown or positive HIV-1 status is an exclusion criterion for all PrEP products		
Clinical criteria	• Weight < 35 kg • Previous hypersensitivity to any components.	• < 18 years old • Pregnancy • Hypersensitivity to any of the excipients (*Dimeticone and/or silicone elastomer)*	• Inflammatory skin conditions that compromise the safety of intramuscular (IM) injections • Weight < 35 kg • Previous hypersensitivity reaction to Cabotegravir
Contra-indicated concomitant medications	Co-administration with other Tenofovir-containing, emtricitabine-containing, or lamivudine containing products	None	(1) Anticonvulsants: Carbamazepine, oxcarbazepine, phenobarbital, phenytoin (2) Antimycobacterials: Rifampicin, rifapentine

Pregnant participants are included in the study and able to select oral or injectable PrEP options, following comprehensive risk-benefit counselling. The safety of oral PrEP in pregnancy is well-established and recent data demonstrates that CAB-LA is well tolerated during pregnancy and postpartum, has a similar pharmacokinetic profile in nonpregnant and pregnant women, and has shown no significant increases in adverse pregnancy or birth outcomes, including neural tube defects [17, 19]. The DapiRing is currently not approved for use in pregnant and breastfeeding women in South Africa but is expected to approved soon with recent research demonstrating safety for use in pregnancy [20]. 

Participants may be naïve to PrEP use, using PrEP through government PrEP services, or they may be recruited from an existing and affiliated large-scale PrEP programme in the study area (“FASTPrEP”), which operates four mobile clinics, a PrEP depot site, as well as from all public health clinic facilities in the study area [21]. FASTPrEP is a novel demonstration project designed to introduce, scale up, and evaluate current (oral PrEP and DapiRing) provision through differentiated models of service delivery that integrates PrEP into a comprehensive SRH package [21]. Enrolment on PtC for non-FASTPrEP participants necessitates simultaneous co-enrolment on FASTPrEP. PtC is promoted at all existing FASTPrEP sites and through its existing social media platforms.

*Sample size calculation*: With 900 participants who start CAB-LA, the study will have > 85% power to detect an 8% or greater difference in PrEP persistence at 7 months comparing oral PrEP users to injectable PrEP or injectable vs. DapiRing selectors using a simple chi-square test. We conducted a sensitivity analysis to estimate the power with different levels of sample size at 95% confidence interval. If a lower sample size were reached, we would have > 80% power to detect a 11% difference or more with 700 participants in CAB-LA vs. 200 in oral PrEP. Study enrolment will cease once a minimum of 900 participants have self-selected to initiate on CAB-LA at baseline, irrespective of the number of oral PrEP and DapiRing users at that time point.

#### PrEP providers and their role

PrEP delivery in SA is nurse-driven and delivered, with PrEP dispensing allowable for any Nurse-Initiated Management of Antiretrovirals (NIMART) qualified practitioner. PtC is delivered by a team of PrEP providers that include NIMART qualified nurses, HIV counsellors, and peer navigators (trained youth aged 18–29 from the study area) [21]. At both the mobile and clinic setting, potential participants are welcomed by peer navigators who provide general HIV health promotion talks and information about the study. Interested participants are seen by the HIV counsellor, following registration and consent procedures, who conduct HIV testing and PrEP choice counselling (average time 30 min) for all HIV negative participants.

Participants are then referred for a nurse-led clinical assessment for eligibility to use the PrEP product of choice and PrEP administration. Administration involves injection of CAB-LA, provision (and insertion if requested) of the DapiRing, or provision of a 30-day oral PrEP supply. All participants are required to return at one month post PrEP initiation to assess safety and interest in continuation on the product and receive their second loading CAB-LA injection or a three-month supply of oral PrEP or DapiRing. All members of the team are trained in adolescent and gender friendly health care and good clinical practice.

#### PrEP choice counselling

 The PrEP choice counselling script was co-created through two formative workshops, one with PrEP-experienced health care providers and administrators (*n* = 25) and one with members of the FASTPrEP youth reference committee (*n* = 10). Feedback from these workshops, alongside choice counselling literature and South African national recommendations for CAB-LA counselling, was used to create the first version of the script, which is delivered using a motivational interviewing approach [22]. The counselling process is monitored through quarterly fidelity checks with opportunity for iterative adjustments based on feedback from the fidelity visits and the counsellors. The aims of the counselling session (which were created during the formative workshops) are firstly, to provide comprehensive information about each PrEP product, and secondly, to empower the PrEP user to contextualise that information within their own lifestyles and sexual practises.

#### Setting

Participants are recruited from the Mitchells Plain-Klipfontein health-sub-district in Cape Town, South Africa. Demand creation for PrEP is currently ongoing in this district under the FASTPrEP programme, whereby the communities are exposed to PrEP educational materials and encouraged to visit community and public health sites to access SRH services, including PrEP [21]. While the FASTPrEP project operates from several mobile, clinical, and community sites (including schools), this study operates from a single mobile and a single clinic site. These sites were selected based on a record of high numbers of oral PrEP users during the preceding year of FASTPrEP implementation and support from local district health officials. The overall model of differentiated PrEP service delivery is outlined in Table 3.

The mobile setting is a Desmond Tutu Health Foundation run mobile clinic that comprises of trained research staff who follow research-specific protocols with resources afforded to them by the research project budget. The primary aim of this site is SRH services and associated research activities. The government health clinic is a government primary health care facility comprising of government clinical and administrative staff, with support from PtC research staff embedded on-site (including a research assistant, HIV counsellor, and two peer navigators), and which follows national, provincial, and local healthcare delivery guidelines and is limited by the resources available to the state. The primary focus of this clinic is healthcare delivery, including services beyond SRH such as antenatal care, HIV treatment, and a midwife obstetric unit. There are minor differences in clinic flows between the sites which have potential to differentially influence the participant experience of the study, PrEP choice counselling, and PrEP administration.

**Table 3:** Differentiated service delivery of PrEP choice

	Oral PrEP	Vaginal PrEP	Injectable PrEP
Where (Service Location)	Mobile clinic Government health clinic		
Who (Service Provider)	NIMART-qualified nurses (with medical officer oversight) Lay counsellors Peer Navigators		
When (Service Frequency)	At initiation – one month supply given After 1 month – 3 month supply given	At initiation – one month supply given After 1 month – 3 month supply given	Initiation doses: At initiation and one month Maintenance doses: Every 8 weeks
What (Service Package)	PrEP Choice counselling HIV testing: self-testing option for courier users. Comprehensive SRH service: STI testing and treatment Pregnancy testing and contraceptive counselling		The same, excluding HIV self-testing option

### Clinical intervention components

PrEP schedules differ by product, as follows:

#### Oral PrEP

Oral PrEP is currently standard of care in SA and this study follows national oral PrEP guidelines with regards to counselling and administration [23, 24]. A notable difference is the promotion of event-driven PrEP for male participants, whereby oral tablets are taken in a 2-1-1 formation (two tablets 2–24 h prior to potential HIV exposure and then continue one tablet daily until two days after the last potential exposure). Event-driven PrEP is not part of national PrEP guidelines but is widely supported and expected to be introduced into updated national guidelines expected in 2025.

#### DapiRing

The DapiRing provides a slow release of Dapivirine, a non-nucleoside reverse transcriptase inhibitor, intra-vaginally for 28 consecutive days [25]. The first ring is inserted by the nurse/clinician with guidance given for future self-removal and self-insertion and ongoing training is provided at replacement visits as required. As DapiRing is becoming part of standard of care in SA, it is available at select demonstration sites outside of this study [25]. 

#### CAB-LA administration

CAB-LA is delivered in a long-acting suspension via an intramuscular injection every eight weeks, as a 3 ml injection containing 600 mg of Cabotegravir [26]. Administration is performed by a NIMART qualified professional nurse trained in antiretroviral drug delivery and in the appropriate injection technique. All sites are equipped with resuscitation equipment and trained in basic life support, with participants required to remain on site for a 10-minute observation period post-injection. A second initiation dose is provided at 1 month. Participants are required to return for that scheduled visit within 28 days in order to continue the schedule. Those that return more than 28 days after the scheduled visit (i.e. >56 days after the first injection) are required to re-start the injection schedule. Thereafter, injections are given every 8 weeks with specific guidelines to support continuation after missed injections (< or > 4 weeks since target injection date) [26]. 

For both oral PrEP and DapiRing, a single month’s supply (one tablet bottle or one ring) is supplied at the initiation visit and the participant is required to return after one month to assess product suitability and sustained use. If the participant selects to continue with this product, they are provided with a three-month supply (three tablet bottles or three DapiRing products). This reduces the overall number of clinic visits required for oral and ring participants compared to injectable PrEP, which necessitates in-person visits at least every 2 months. Oral and ring PrEP users may further opt to receive their next supply via courier. In the courier package, they receive an HIV self-test, instructions on how to do the test and where to send results. Access to subsequent courier deliveries is dependent on disclosure of HIV self-testing results.

Participants are counselled to return for study visits even if they would like to discontinue PrEP such that they can receive support in switching PrEP products or safely discontinuing. PrEP product switches are allowed at any point throughout the 18 month study timeline. Written material on safe discontinuation is provided and a WhatsApp line for PrEP support is available if they are unable to return. An automated reminder is sent by short messenger service (SMS) one week prior to the scheduled visit. Participants that do not return for scheduled visits within 7 days are followed up via phone, message, and home visit (if safety is a concern and permission has been granted). Specific counselling on the duration of CAB-LA in the body post injection and the potential risk of antiretroviral drug resistance developing if HIV infection occurs is included before and after starting CAB-LA.

### Clinical testing and screening

#### HIV testing

 All participants are required to undergo HIV testing prior to commencement with PrEP products to confirm their HIV-negative status. There is a risk that participants initiated on CAB-LA during an acute HIV infection will have a delayed immune response to HIV (suppressing HIV viral replication and thereby reducing the generation of anti-HIV antibodies- this is known as the LEVI syndrome) [27]. This could delay diagnosis and increase the risk of HIV drug resistance. To mitigate this risk, some regulatory bodies have required NAAT HIV testing at baseline and throughout CAB-LA to allow for early detection. The WHO, however, have acknowledged the high cost and resource burden of NAAT testing as well as its lack of regulatory approval as an HIV diagnostic test and recommends continued use of national HIV testing algorithms that rely on 3rd or 4th generation rapid antibody/antigen tests. To investigate this approach, PtC participants undergo HIV testing at each visit with 3rd and 4th generation HIV rapid tests, the results of which guide eligibility for same day PrEP initiation, after additionally confirming the absence of signs or symptoms of acute HIV exposure and/or a recent history of a potential HIV exposure that would indicate the need for post-exposure prophylaxis (PEP). Viral load (NAAT) testing is conducted in the laboratory at baseline, month 1, month 7 (range 5–8 months), and month 17 (range 16–18 months) post-PrEP initiation to validate the rapid results. In the case of a detectable viral load test result post-PrEP initiation, the participant is recalled for further confirmation of the HIV testing results and rapid transition to HIV treatment and care if applicable. This will provide evidence to confirm or refute the superiority of (and thus necessity to use) more expensive and resource-intensive viral load tests for CAB-LA users in this setting. Participants on CAB-LA who seroconvert are invited to take part in an affiliated study investigating salvage treatment options.

#### Additional safety tests

 At study initiation, all CAB-LA selecting participants have blood draws to screen for Hepatitis B (Hepatitis B surface antigen test) and an abridged liver function test (including liver transaminases and total bilirubin) to rule out pre-existing liver pathology. CAB-LA injections are not delayed until these results are available and participants are recalled within a week for clinical review should any laboratory results be of concern. In the case of concerning results, participants are recalled and managed on a case-by-case basis with oversight from the study’s Clinical Management Team. Previous work in this study setting showed that HBV prevalence is very low in young female populations (0.69% overall and 0% in those born after 1995), likely due to high HBV vaccine coverage; however, it may be of concern in older male participants that are also at greater list of other hepatology conditions and greater alcohol use [28]. 

#### Sexual and reproductive health package

PtC participants are offered a comprehensive SRH package of services, in line with those described for the FASTPrEP study [21]. These services include family planning (pregnancy testing, contraceptive counselling, and contraceptive administration) and testing or screening (resource-dependent) and treatment for sexually transmitted infections (*Chlamydia trachomatis*,* Neisseria gonorrhoea*,* and syphilis).* All participants are further screened for alcohol misuse (AUDIT scoring system), mental health (Patient Health Questionnaire (PHQ-4), and tuberculosis (TB) symptoms. Referral pathways are in place following positive HIV or pregnancy tests, positive TB screening, detection of intimate partner violence, and for high AUDIT or PHQ-4 scores.

### Data collection, safety, and ethical oversight

Potential participants are invited to undergo an informed consent process, that covers their participation in both the FASTPrEP project and the PtC study. As this study concerns use of PrEP as part of SRH services, participation assumes ongoing sexual activity and all PrEP products and SRH services offered are approved for use and the majority are available in public healthcare settings for minors (< 18 years). Since this is health research, participation amongst minors (aged 15–17 years old) is usually restricted by the need for parental/guardian consent. Given the need to reduce barriers to participation a parental consent waiver has been obtained and is available for participants aged 15–17 years old that allows them to self-consent for participation in the study. Parental involvement and disclosure is encouraged.

Potential participants are screened for co-enrolment in other HIV related clinical trials in the area using the nationally accredited Biometric Co-Enrolment Prevention System (BCEPs), which requires biometric registration. Once co-enrolment is excluded, participants have their biometrics registered on a separate participant tracking system that creates a unique participant profile and allows participants to move freely between PtC/FASTPrEP service sites [21]. 

All study data, including the informed consent forms, is captured directly onto an online REDCap system by study staff (including recruiters, counsellors, clinicians, and research assistants) at the time of data reporting. Demographic, behavioural (including sexual behaviour), and clinical information (including medical history, obstetric history where applicable, use of sexual and reproductive health services, HIV testing, and PrEP use) are collected at each study visit. Point of care and laboratory results are captured as they become available to the unique participant REDCap profile. A return visit auto-calculator that can calculate the date of return for PrEP continuation is built into the REDCap database. This calculator allows for manual adjustment to account for participant schedules and holidays and aids clinicians in accurately informing participants of their return dates.

PrEP is provided by NIMART nurses at the study site, while all safety blood results (hepatitis B, liver function tests, and HIV NAAT test results) are delivered electronically to the study’s medical officer (PM) for review. The medical officer is available at all times for site staff to contact telephonically if there is a need for clinical review. All study staff are trained on basic life support and a resuscitation trolley is available at both mobile and clinic sites. All sites have established referral pathways for medical concerns, mental health needs, and in case of disclosure of intimate partner violence. Clinical safety data is reviewed monthly by a dedicated clinical management team that includes three clinicians, two of which are infectious disease specialists, and other senior study investigators. The clinical management team includes Desmond Tutu Health Foundation (the sponsor) staff, which both investigators from the PrEPared to Choose study and experienced clinicians and researchers not involved in the study.

The study team includes a dedicated internal monitor who on a continuous basis performs monitoring duties as per International Council for Harmonisation Good Clinical Practise guidelines. Quality assurance (QA) and regulatory audits are conducted at random by the sponsor’s independent QA and Regulatory department. Audit feedback is provided in writing and filed in the investigator site file. The study is subject to external audits by SAHPRA at any time. Six monthly progress reports and annual study reports are sent to SAHPRA. Annual re-approval is submitted to the ethics committee. Bi-annual feedback on the study is provided to ViiV Healthcare and the Bill and Melinda Gates Foundation. Planned dissemination activities for this study include the submission of abstracts to local and international conferences, submission of manuscripts to peer-reviewed journals, and community dissemination events at study end. The youth reference group engaged for this study and for the FAST PrEP platform is regularly updated around study progress, study materials, and study challenges.

### Study reimbursement

To simulate a real-world environment where PrEP is delivered within a standard service of care from public health care facilities, no reimbursement for inconvenience (such as time spent or transport) is offered to participants who participate in this study. The SRH service package and PrEP modalities are, with the exception of CAB-LA, part of the existing public healthcare service in SA. Participants that are invited to and attend research visits for additional questionnaires, in-depth interviews, and focus group discussions are provided with R150 ($8.50) per visit. No post-trial access to injectable PrEP product is provided at the end of the study, however, the study team is committed to advocating for the incorporation of injectable PrEP products into standard of care services. Oral and DapiRing PrEP products are already available for free through standard of care service provision in government clinics. At the end of the study, participants that remain interested in PrEP use will be transitioned into the public healthcare PrEP service. Clinical trial insurance has been obtained.

#### Statistical analysis

PrEP initiation in this study is defined as having received a 28-day regimen of oral PrEP tablets, receiving a DapiRing, or receiving a CAB-LA injection depending on the respective product choice. Initiators are participants that have selected a specific PrEP product at baseline after receiving PrEP choice counselling.

To determine the primary and secondary endpoints of short-term and durable persistence (as defined above), a Cox proportional hazards model to estimate the hazard of discontinuing PrEP at 7 months and through 18 months will be conducted, thus taking into account the presence of censoring due to end of study period or drop-out, including all participants who are confirmed as HIV-uninfected at enrolment and who attend at least the PrEP initiation visit (visit 1).

More specifically, PrEP persistence at 7 and 18 months will be summarized in the first instance as proportions of subjects on a given modality at those time points out of those who started on the modality and compared using a chi-square test. Additionally, Kaplan-Meier curves will illustrate the time to discontinuation by PrEP modality. Cox proportional hazards model will be used to analyse the risk of discontinuation overall, (i) regardless of modality and (ii) taking modality into account. Frailty models will account for repeated discontinuations per subject. Modality will be included as a time-varying exposure variable. Additionally, time to switching modality will be analysed using a Cox proportional hazards model, with frailty terms to account for repeated switches, if required. Models will include subject-specific characteristics that may confound the association between modality and risk of discontinuation. Different subgroups (adolescent girls and young women, intimate male partners, and gay and other men who have sex with men) can be included as a predictor in the models and additionally analysis will be repeated for sub-groups of interest. The average time to restart, the mean number of restarts for a participant and the total number of restarts across the study period will be reported and used to describe the variation in PrEP user journeys throughout the product.

Deeper understanding of the PrEP user experience will be drawn from self-administered questionnaires (n = up to 180 randomly selected participants at month 0, 6, and 12) and in-depth interviews (IDI) with a sub-set of PrEP providers and PrEP users. Survey data will be summarised and described. IDI’s will be audio-recorded, translated (with independent back-translation for verification), and transcribed for analysis. The recordings will be reviewed and verified for accuracy by the interviewers. Transcripts will then be translated and key quotations back-translated to verify meaning. A subset of 20% of transcripts will be double-coded to examine the inter-rater reliability of the coding process. After coding, investigators will search by substantive codes and themes identified utilising matrices to organize and allow for comparisons by group (e.g., PrEP users: age, PrEP product selected at baseline, PrEP product switching, partner status; and PrEP provider: age, site of PrEP distribution, PrEP product administered). Patterns in the matrices will be used to identify themes. Tables of themes will be created using the verbatim quotations as evidence to support the themes that serve as the primary data analyses approach. Conclusions will be based on interpretation of these tables of themes with quotations. Data will be managed using NVivo Software (QSR International). Changes in PrEP user and PrEP provider perspectives over the 6-month period (collectively and across matched individual) will be quantified to identify changes pre- and post- CAB-LA experience at the user or provider level, with McNamara’s test for matched pairs used to determine significance.

## Discussion

The PtC study is evaluating the real-world implementation of PrEP choice, with the first-time inclusion of three approved PrEP products. The protocol was specifically designed to simulate a realistic context for PrEP roll-out, with the aim to firstly, evaluate persistence with expanded PrEP choice and secondly, describe practical challenges that may occur when recruiting young people into HIV prevention services, providing PrEP choice counselling, initiating PrEP, and supporting PrEP continuation.

These aims are supported by the inclusion of two types of settings, mobile clinics and primary health care facilities. Mobile clinics are capable of reaching participants in community spaces (such as malls, sports grounds, and transport hubs), offer a tailored and efficient health services, and can be designed to be adolescent friendly. These attributes partially overcome some of the well-described challenges of primary health care clinics [21]. However, mobile services in this study are also staffed by teams that may be better resourced (in terms of staff, equipment, and support) and more familiar with research than in clinic settings. This may falsely elevate PrEP outcomes in this setting, compared to a more traditional government setting. The government primary health care facilities are small clinics with limited resources and staffing, that provide a limited primary care service that includes SRH and HIV treatment and care. Some of the barriers to services in this environment have been reported to include long waiting times, unfriendly staff attitudes, high staff turnover, and medication stock outs [1, 3]. However, the vast majority of PrEP choice in SA will be delivered from such facilities which therefore provide a realistic view of PrEP implementation challenges.

PtC has purposefully included a broad population range of adolescent and young people, including heterosexual males and gender and sexually diverse populations, rather than tailoring the service to adolescent girls and young women (a traditional PrEP target in the Eastern/Southern African setting). This was pursued intentionally to firstly, reduce stigma that can arise from only offering PrEP to a single population designated to be high risk, and secondly, to allow for a description of natural PrEP uptake rates among different populations when offered as a generalised service to young people. The resulting data will be used to inform which populations can be accessed through generalised services and which populations may need dedicated outreach interventions and spaces. Where possible, peer navigators from sexual and gender diverse groups have been employed.

The introduction of injectable PrEP, CAB-LA, has been broadly anticipated to alter the PrEP landscape, increasing both uptake and persistence. However, CAB-LA use up until this point has only been described in the context of clinical trials where robust eligibility criteria, study reimbursement, and strict visit schedules likely influenced the rate of on time return for subsequent injections. On time return in these studies was described as within a 7-day window [17, 18]. In the South African context, this is broadly considered to be unrealistic due to several factors including participant challenges with transport, inflexible work schedules, and community safety concerns. It is hypothesized that a more flexible 28-day visit window is likely to allow an improved rate of return and persistence, while still operating within the bounds of clinical safety and PrEP product efficacy. The PtC study as such will report on persistence using both a 7 day and 28-day visit window to highlight the impact of this on reported persistence outcomes.

A primary focus of this study will be to develop a feasible and acceptable means of PrEP choice counselling delivery that ensures accurate information while supporting the agency of the individual PrEP user. One challenge is that choice counselling will occur in a busy clinic environment necessitating a rapid and easy to use strategy. Another challenge is the concise and accurate communication of new and unfamiliar concepts with newer PrEP products, such as the long duration of injectable PrEP and the association with antiretroviral drug resistance or the placement, comfort, and daily impact of having a DapiRing in situ.

## Limitations

This study has several limitations. Firstly, while an extensive effort will be made to simulate real-world conditions, there are some aspects of the clinical trial setting that cannot be avoided. This includes oversight from highly qualified and trained study staff members that may offer a higher level of specialized care than what is provided in public healthcare settings where staff turnover may be high. PtC staff members are specifically trained in adolescent and youth friendly service provision and will undergo gender sensitization training, which is not necessarily part of standard of care training packages. Secondly, the PtC counselling intervention assumes the continuous availability of an experienced HIV counsellor, which is not always the case in primary healthcare settings where counsellors may also often see high staff turnover. Thirdly, the mobile clinic and government primary health clinic site are likely to have notable differences including the number of study support staff and the focus of the service, with the mobiles offering a targeted SRH service and the clinic supporting multiple diverse primary care services. Outcomes that may be influenced by these differences will be formally tested for in the study analysis.

## Conclusion

South Africa benefits from being one the first countries to receive regulatory approval of and access to all currently available PrEP products. HIV prevention services, including HIV testing and counselling and oral PrEP provision, is already well-integrated into primary health care with antiretroviral-based medication (including treatment and PrEP) prescribed by trained and accredited primary care nurses. There is broad public awareness of HIV, with appreciation for the high HIV prevalence and incidence and increasing general interest in HIV prevention services. Yet, oral PrEP has been available in SA for more than a decade, and although SA has been a leader in numbers of new PrEP users in 2024, initial implementation was hindered by slow roll-out and lower than expected uptake and persistence. PtC is an implementation project that will contribute to understanding how implementing PrEP choice may enhance uptake and effective use. This will inform future efforts to implement PrEP choice rapidly, safely, and at a scale sufficient to drive down HIV transmission in South Africa.

## Data Availability

No datasets were generated or analysed during the current study.
